# Clinical efficiency of simultaneous CNV-seq and whole-exome sequencing for testing fetal structural anomalies

**DOI:** 10.1186/s12967-021-03202-9

**Published:** 2022-01-03

**Authors:** Xinlin Chen, Yulin Jiang, Ruiguo Chen, Qingwei Qi, Xiujuan Zhang, Sheng Zhao, Chaoshi Liu, Weiyun Wang, Yuezhen Li, Guoqiang Sun, Jieping Song, Hui Huang, Chen Cheng, Jianguang Zhang, Longxian Cheng, Juntao Liu

**Affiliations:** 1 Department of Ultrasound Diagnosis, Maternal and Child Health Hospital of Hubei Province, Wuhan, 430070 Hubei China; 2 Department of Obstetrics and Gynecology, State Key Laboratory of Complex Severe and Rare Diseases, Peking Union Medical College Hospital, Chinese Academy of Medical Sciences and Peking Union Medical College, Beijing, 100730 China; 3 Berry Genomics Corporation, Beijing, 102200 China; 4 Department of Obstetrics, Maternal and Child Health Hospital of Hubei Province, Wuhan, 430070 Hubei China; 5 Department of Genetic Laboratory, Maternal and Child Health Hospital of Hubei Province, Wuhan, 430070 Hubei China; 6 Department of Ultrasound Diagnosis, Hubei Maternity and Child Health Hospital, No. 745, Wuluo Road, Hongshan District, Wuhan, 430030 Hubei China; 7 Department of Obstetrics and Gynecology, Peking Union Medical College Hospital, Peking Union Medical College and Chinese Academy of Medical Sciences, No. 1, Shuaifu Garden, Dongcheng District, Beijing, 100730 China

**Keywords:** Whole-exome sequencing, Prenatal diagnosis, CNV-seq, Structural anomaly

## Abstract

**Background:**

Birth defects are responsible for approximately 7% of neonatal deaths worldwide by World Health Organization in 2004. Many methods have been utilized for examining the congenital anomalies in fetuses. This study aims to investigate the efficiency of simultaneous CNV-seq and whole-exome sequencing (WES) in the diagnosis of fetal anomaly based on a large Chinese cohort.

**Methods:**

In this cohort study, 1800 pregnant women with singleton fetus in Hubei Province were recruited from 2018 to 2020 for prenatal ultrasonic screening. Those with fetal structural anomalies were transferred to the Maternal and Child Health Hospital of Hubei Province through a referral network in Hubei, China. After multidisciplinary consultation and decision on fetal outcome, products of conception (POC) samples were obtained. Simultaneous CNV-seq and WES was conducted to identify the fetal anomalies that can compress initial DNA and turnaround time of reports.

**Results:**

In total, 959 couples were finally eligible for the enrollment. A total of 227 trios were identified with a causative alteration (CNV or variant), among which 191 (84.14%) were de novo. Double diagnosis of pathogenic CNVs and variants have been identified in 10 fetuses. The diagnostic yield of multisystem anomalies was significantly higher than single system anomalies (32.28% vs. 22.36%, P  = 0.0183). The diagnostic rate of fetuses with consistent intra- and extra-uterine phenotypes (172/684) was significantly higher than the rate of these with inconsistent phenotypes (17/116, P  = 0.0130).

**Conclusions:**

Simultaneous CNV-seq and WES analysis contributed to fetal anomaly diagnosis and played a vital role in elucidating complex anomalies with compound causes.

**Supplementary Information:**

The online version contains supplementary material available at 10.1186/s12967-021-03202-9.

## Background

Birth defects are responsible for approximately 7% of neonatal deaths worldwide by World Health Organization in 2004 [1]. The incidence of birth defects in high-income countries is 4.7%, while that in the middle-income and low-income countries is 5.6% and 6.4%, respectively [1]. Based on a recent survey in China mainland, the prevalence of birth defects is substantially 4–6% [2]. Ultrasonography plays a vital role in birth defect screening in the prenatal stage, as it contributes to about 3% of fetal structural anomalies during prenatal ultrasound screening [3].

Karyotype and chromosomal microarray analysis (CMA) have been commonly utilized for examining the congenital anomalies in fetuses [4, 5]. Karyotype could identify aneuploidy, translocation, and inversion of genome. Likewise, microarray could detect submicroscopic CNV5. The prevailing diagnostic yield for karyotype was 32% among the fetuses with abnormal ultrasound findings [5, 6]. For the fetuses with no abnormalities after karyotyping analysis, the integration of CMA yielded an extra detection rate of 3–5% [4]. Recently, next-generation sequencing (NGS) has been developed as an alternative method for detecting CNV [7–9]. In a recent large invasive CNV-seq cohort conducted by our team, CNV-seq provided a high reliability and accuracy for identifying clinically significant CNVs relating to fetal anomalies in prenatal samples [10].

Recently, whole-exome sequencing (WES) has been gradually utilized in clinical settings for the diagnosis of certain diseases suspected to be monogenic or oligogenic [11, 12]. WES has been proved to be a feasible tool in prenatal diagnosis as it can detect single-nucleotide variant (SNV), small insertion/deletions (InDel) and CNVs covering multiple exons [13–16]. Accordingly, exonic CNVs could induce genetic diseases with the involvement of SNV/InDel in a trans-phase [15, 17]. WES leads to a diagnostic yield of 8.5–10% for fetal structural anomalies in those with negative findings after karyotype analysis and CMA [13, 14]. A sequential karyotype, CMA (or CNV-seq) and exome sequencing (ES) strategy was widely approved and performed in the prenatal anomaly clinical setting. When cases following this sequential strategy were detected a pathogenic CNV, this workflow would be stopped and the ES would not be performed that the information of genetic variants was missed. However, more and more double diagnosis, and even triple diagnosis, has been revealed in prenatal and pediatric cases. The missing genetic variant information might lead to a misdiagnosis or inadequate of following health care.

With the advances of ultrasound technique, ultrasonography has been reported to detect structural anomalies at an extreme gestational stage, such as 11 weeks or end-stage of pregnancy [18, 19]. Prior study revealed that ultrasound scanning contributed to the screening of fetuses (33.35%) with structural anomalies in the third trimester nowadays [18]. The sequential karyotype-CMA-ES strategy might face with its limitation when performing on these cases. Due to inadequate amount of sampling in the early gestational stages, extracted DNA would not be able to afford a complete sequential test. Furthermore, routine karyotype-CMA-ES strategy requires a long time period (up to 50 days). Therefore, a more rapid turnaround time (TAT) of test pipeline is urgently demanded and would provide a higher quality genetic counseling in the restricted time window, particularly when the ultrasound anomalies were detected at the late stages [20, 21].

In our previous study, a simultaneous CNV-seq and WES strategy has been established to meet the impending requirements for the diagnosis of congenital defects [22]. However, the cohort was limited in sample size, and a comprehensive evaluation based on a large sample size is crucial. In this study, simultaneous CNV-seq and WES is conducted based on an experimental optimization and data integrated method for 959 Chinese trios with congenital defect fetuses. We aimed to comprehensively elucidate genetic alteration of the fetal defect and evaluate the efficiency and benefits of simultaneous CNV-seq and WES analysis.

## Methods

### Study design and participants

This retrospective study was performed in a tertiary level referral center, the Maternal and Child Health Hospital of Hubei Province (MCHHHP). The study protocols were approved by the Medical Ethics Committee of MCHHHP. Pregnant women confirmed after ultrasound at local hospitals over 11 gestational weeks in Hubei Province were recruited in this study. Couples of singleton fetus with structural anomalies or increased nuchal translucency (NT) were eligible. Routine procedures for prenatal genetic diagnosis were performed in local hospitals. Couples of fetuses diagnosed as aneuploid were excluded.

Eligible pregnant women were transferred to the MCHHHP through a referral network. Written informed consent was obtained from each couple. Then demographic characteristics were recorded through a questionnaire containing maternal and paternal age, history of gravidity and parity, consanguinity, history of abnormal pregnancy and reproduction, pregnant naturally or in vitro fertilized, medical history, as well as family history of inherited diseases. Couples receiving blood transfusion within 1 month were excluded.

Prenatal ultrasonic results were re-scanned by staff sophisticated in prenatal ultrasonography in MCHHHP. Scanning was in line with the practice guidelines proposed by International Society of Ultrasound in Obstetrics and Gynecology (ISUOG) [23–25]. Quality control of the ultrasound scanning was conducted in line with a unified standard in the MCHHHP. Findings were collected and managed in an in-house database. Couples with their fetuses confirmed as structural anomalies or increased NT were included. Afterward, multidisciplinary consultation was carried out by at least two senior physicians in obstetrics and ultrasonography, respectively. With the advice after multidisciplinary consultation, couples were well informed about the fetal phenotype and then made their decision on fetal outcome at MCHHHP or local hospitals. Finally, qualified couples were required to provide a products of conception (POC) sample after fetal outcome, and parental peripheral blood was collected for trio analysis. Mother-father-fetus trios with incomplete parental samples were excluded. Samples were collected in MCHHHP or local hospitals and were all processed and preserved in MCHHHP for further test.

### Procedures

We recruited 1800 pregnant women in Hubei Province between June 2018 and October 2020 underwent prenatal ultrasound screening. After multidisciplinary consultation and decision on fetal outcome by the parents, we then obtained POC samples including umbilical cord section, umbilical cord blood, placental sections, and the tissues after abortion. Samples were all stored at − 20 °C for the following experiments. During the sampling process and genetic testing, parents were informed about the research purposes. Only confirmed causative results would be reported, and TAT was 10–14 days.

DNA was extracted from trio samples by MagMAX DNA Ultra 2.0 (Thermo Fisher, CA, USA). Then DNA samples were sequenced on the NGS platform (Berry Genomics, Beijing, China). PCR-free-frag library was constructed for CNV-seq, with our unique experimental pipeline previously described26. Briefly, genomic DNA (10–40 ng) was treated (NEBNext dsDNA Fragmentase, New England Biolabs, Ipswich, MA, USA) and inputted into the experimental system (KR2000, Berry Genomics, Beijing, China) to generate library for sequencing. Approximately 5 million 37 bp plus 8 bp (index) raw reads were generated for each sample after library sequencing on the NextSeq CN500 platform (Berry Genomics) with a run time of 6.5 h. Concurrently, initial genomic DNA (50 ng) was whole-exome captured depending on custom-designed probe NanoWES (Berry Genomics, Beijing, China). Library preparation was performed using Human Whole Exome Detection Kit (Berry Genomics, Beijing, China), and HiFi HotStart ReadyMix (KAPA) was used for library amplification. The amplicons were subject to paired-end sequencing on a NovaSeq 6000 platform (Illumina) with the paired-end 150 bp protocol.

Raw reads generated after CNV-seq were edited to remove artificial adaptor sequences. Then the processed sequences were mapped to the GRCh38 reference genome, which was conducted by the Burrows-Wheeler Alignment tool (version 0.7.5a). Reads were processed and CNVs were evaluated by an in-house pipeline using read counts based on a smoothness model (Berry Genomics, Beijing, China) according to the previous description [26]. In brief, processed reads were divided into continuous 20 kb bins. The first-order difference was then performed to regularize the read counts of the N (from the first bin to the end of a chromosome) and N-1 bins. This regularization was conducted by a dynamic processing approach using all data from the same batch. A smoothness model based on a regression calculation was then processed on the regularized data. After those processing above, the location where first-order difference was still not zero would be identified as a potential CNV breakpoint. Continuous two breakpoints were marked as a potential CNV for furthering manual examination.

Adapters and low-quality sequences in raw reads of WES were simultaneously removed by Flexbar (version 3.5.0). Processed reads were aligned to the GRCh38 reference genome using the Burrows-Wheeler Alignment tool (version 0.7.5a), which was sorted and marked duplications by Sambamba (version 0.7.0). Genetic variant calling was performed by Strelka (version 2.9.10), and variant quality was screened based on the following criteria: Genotype Quality score  ≥ 15; fraction of low-quality bases at a site  ≥ 0.4; and variant read depth  ≥ 3. A previous algorithm (XHMM, v1.0 [27]) was applied to call exonic CNVs. At least three continuous exons consistent with the CNV calling criteria of XHMM were marked as a potential exonic CNV. These genomic variants and CNVs of a parent-fetus trio were integrated into a file with our in-house pipeline for further analysis.

Genomic variants were annotated by Ensembl Variant Effect Predictor (version 102.0), and CNVs were annotated by an in-house pipeline that was published before [26]. We manually transferred the ultrasound findings into Human Phenotype Ontology (HPO) terms. Hence, a published phenotypic scoring algorithm Phrank [28] based on HPO terms was employed to assist in prioritizing alterations. Annotated variants and CNVs were then prioritized and filtered according to phenotype relativity, inheritance mode, allele frequency, read depth, literature, and in silico prediction. Furthermore, candidate variants and CNVs were compared to the reports in the latest ClinVar, ClinGen, DECIPHER, DGV, Human Gene Mutation Database and Online Mendelian Inheritance in Man. The interpretation of pathogenicity was then considered in the context of the American College of Medical Genetics and Genomics (ACMG) guidelines [29, 30]. Only pathogenic, likely pathogenic variants and CNVs would be reported. Report pipeline of incidental findings was consistent with the ACMG recommended list [31]. The incidental findings were not routinely reported to the parents unless an explicit requirement by themselves.

The results were presented to a multidisciplinary conference held by clinical and molecular geneticists, obstetricians, genetic counselors, pediatricians, prenatal ultrasonic experts, and bioinformatics specialists. Genomic variants were confirmed by Sanger sequencing through 3730xl system (ABI). Ambiguous CNVs were confirmed through Infinium Global Screening Array SNP-array (Illumina) and Single Molecule Real-Time sequencing (PacBio).

### Outcome

The primary endpoint assessed in all fetuses were diagnostic genetic variants and CNVs considered to induce fetal developmental anomaly. We also assessed the pre-specified exploratory endpoint by comparing the simultaneous CNV-seq and WES analysis and the sequential karyotype-CMA-ES test strategy.

### Statistical analysis

The number of diagnostic alterations in different phenotypic classes was compared with Fisher’s exact test. All statistical analyses were performed with GraphPad Prism (version 8.0) and Origin (version 9.0). A *P* value of less than 0.05 was considered to be statistically significant.

## Results

### Demographic characteristics

**Fig. 1:**
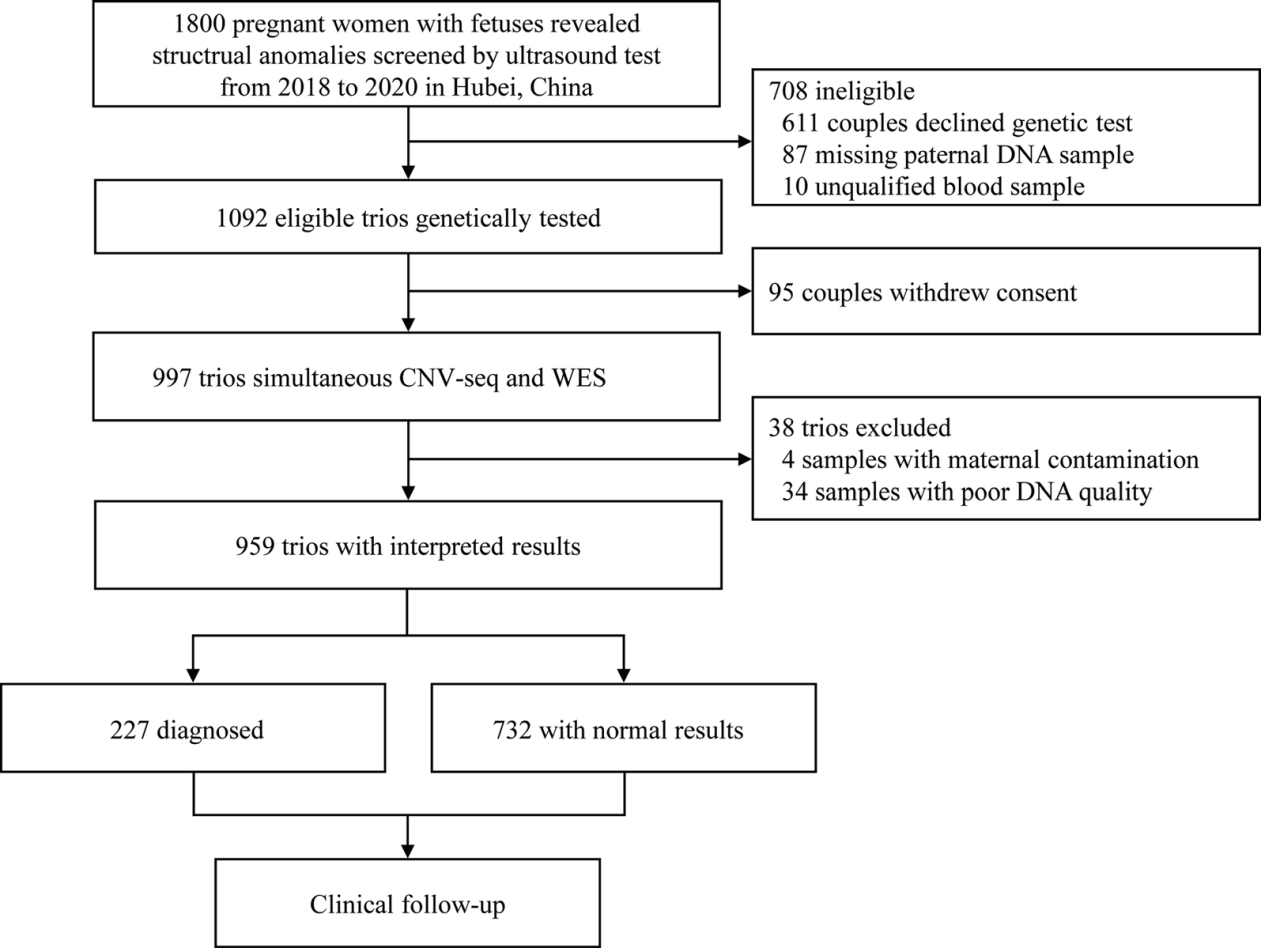
Flow chart of the study design. The exclusion of the present cohort was based on the sample quality, signing informed consent, and extracted DNA quality

Between June 2018 and October 2020, a total of 1800 pregnant women with fetuses showing structural anomalies screened by ultrasound were accessed for eligibility (Fig. 1). Initially, 708 trios were excluded due to decline of any genetic tests (n  = 611), missing paternal DNA samples (n  = 87), and unqualified parental blood samples (n  = 10). Ninety-five couples refused to continue this test, and then were excluded from this study. On this basis, 997 trios decided to undergo simultaneous CNV-seq and WES analysis, and finally 959 trios (male: 557; female: 402) were eligible after excluding 38 cases with maternal contamination (n  = 4) and poor DNA quality (n  = 34) (Fig. 1, Additional file 1: Figure S1).

The enrolled 959 fetuses were categorized into 10 phenotypic classes based on fetal structural anomalies uncovered by ultrasound testing (Additional file 3: Table S1). The phenotypic classes included cardiac, chest and respiratory tract, central nervous system (CNS), facial, gastrointestinal tract and abdominal wall, genitourinary, hydrops, increased NT, skeletal, and complex multisystem anomalies.

Demographic characteristics of trios were collected. The ratio of fetuses of the chest and respiratory tract subgroup was 49% (male) to 51% (female), which was the only subgroup that included more female fetuses (Table 1). The median gestational week for the first screening of fetal structural anomaly was 23.6 weeks. The median gestational week of CNS anomaly was the latest among all classes (26.45 weeks, Table 1). The median paternal and maternal age was 28 and 32 years, respectively (Table 1).

**Table 1:** Demographic characteristics of the participants in this study cohort

	Cases (trios)	Fetal sex (male:female)	Gestational week (weeks)	Maternal age (years)	Paternal age (years)
Total	959	0.58:0.42	23.6 (22.25–26.4)	28 (26–31)	32 (29–34)
Cardiac	265	0.60:0.40	24 (22.6–24.6)	28 (26–31)	32 (30–35)
Chest and respiratory tract	43	0.49:0.51	24.1 (23.15–26.15)	28 (26–30)	31 (30–33.5)
CNS	116	0.53:0.47	26.45 (23.075–31.2)	28.5 (26–31)	32 (29–35)
Facial	127	0.69:0.31	23.2 (22.4–24.35)	28 (25.5–30)	31 (29–34)
Gastrointestinal tract and AW	42	0.57:0.43	24 (14.5–28.125)	29 (27–30)	32 (30–34)
Genitourinary	94	0.62:0.38	24.3 (23–29.45)	29 (27–31)	32 (29–34)
Hydrops	31	0.58:0.42	24.2 (21.5–30.5)	28 (25–31)	32 (28–35)
Increased NT	20	0.60:0.40	13 (12.5–13.3)	28 (27–30)	31 (30–34)
Skeletal	94	0.53:0.47	23.8 (22.15–25.975)	29 (26–32)	32 (30–35)
Multisystem	127	0.54:0.46	22.6 (14.45–24.25)	28 (25–30)	31 (29–33)

Fetuses were counted once. Data were shown as median (first quartile to third quartile)

*CNS* central nervous system; *AW* abdominal wall; *NT* nuchal translucency

### Sampling

POC samples were obtained upon confirmation of fetal outcome. Fetal DNA was extracted from these samples, including umbilical cord segments at birth (n  = 667, 69.55%), tissue samples after pregnancy termination (n  = 182, 18.98%), placental sections (n  = 106, 11.05%), and cord blood at birth (n  = 4, 0.42%, Table 1).

### Diagnostic yields

In practice, candidate 345 CNVs and 4701 genetic variants derived from 284 fetuses were prioritized, among which 17 fetuses were identified with a compound heterozygous state involving CNVs and genetic variants. After interpretation and confirmation by the multidisciplinary conference, pathogenic or likely pathogenic CNVs (n  = 109) and variants (n  = 128) were reported in 227 fetuses (Table 2; Additional file 4: Table S2). Among these 227 fetuses, 10 were identified with a double diagnosis, a causative CNV and a causative variant (Table 2), in which 2 were in a compound heterozygous state involving CNVs and genetic variants (Table 2). Among the other 217 fetuses with single diagnosis, 191 fetuses were de novo including 99 CNVs and 92 genomic variants. Ten male fetuses were X-linked maternal inherited variants. In addition, 2 compound heterozygous genotypes and 8 homozygous genotypes were identified, which were all inherited from both parents. Nine were inherited in an autosomal dominant way from a previously undiagnosed parent.

**Table 2:** Distribution of diagnosis across the anatomical systems of fetuses in the present cohort

	Cases (trios)	Double diagnosis^a^	CNV	Genetic variants	Diagnostic rate (%)
Cardiac	265	3	30	38	26.79
Chest and respiratory tract	43	0	3	1	9.30
CNS	116	0	8	11	16.38
Facial	127	1	7	9	13.39
Gastrointestinal tract and AW	42	1	5	2	19.05
Genitourinary	94	0	6	7	13.83
Hydrops	31	2	3	3	25.81
Increased NT	20	0	3	4	35.00
Skeletal	94	3	9	27	41.49
Multisystem	127	0	25	16	32.28
Total	959	10	99	118	23.67

Fetuses were counted once

*CNV* copy number variation; *CNS* central nervous system; *AW* abdominal wall; *NT* nuchal translucency

^a^Double diagnosis: fetuses that were diagnosed harboring causative CNV and genetic variants (single nucleotide variants and small insertion or deletion)

In the present study, 832 fetuses showed single anomaly as revealed by ultrasound imaging, among which 186 (22.36%) were finally diagnosed with CNVs or genetic variants (Table 2). Among the 832 fetuses, the diagnostic rate was in a range of 9.30–41.49%, which was varied by the categories. The proportion of double diagnosis in fetuses with hydrops (6.45%) was the highest in the single anomaly phenotypic classes (Table 2). There were 127 fetuses with multisystem anomalies, among which 41 (32.28%) were found to have a diagnostic alteration (Table 2). The diagnostic rate of multisystem anomalies was significantly higher than that of the single anomaly (Fisher’s exact two-tailed test, *P*  = 0.0183; Table 2).

### CNV and variant consequence

In this section, we focused on the consequence of the detected CNVs and variants. Among 109 reported pathogenic CNVs, 15 were exonic CNVs, which were identified by continuous abnormal exon reads, ranging from 1.75 to 74.35 kb (Additional file 4: Table S2). Among all 109 fetuses diagnosed with CNVs, 30 fetuses (27.5%) were diagnosed with microdeletion or microduplication syndrome (MMS), and 79 fetuses (72.5%) harbored CNVs postulated to modulate consensus coding regions.

Among all 128 reported pathogenic variants, 75 were missense, 19 were truncating variants, 14 were intronic or synonymous variants that manipulated splice region and impacted pre-mRNA splicing based on in silico prediction, and 20 caused other consequences (Additional file 4: Table S2). All 4 *CHD7* mutations in fetuses were truncating variants, which validated the prior data of typical CHARGE syndrome caused by *CHD7* variants (Additional file 4: Table S2). Among all 128 fetuses with genomic variants, 58 (45.3%) were diagnosed with a syndrome, while the other 70 (54.7%) were diagnosed with single gene disorder (Additional file 4: Table S2).

Distribution of diagnostic yield in distinct categories were divergent. Exonic CNVs were diagnosed most frequently (5.00%) in fetuses of increased NT (Additional file 2: Figure S2). However, the highest diagnostic yield of other CNVs (non-exonic) was 19.69% in fetuses of multisystem anomalies (Additional file 2: Figure S2). Syndromic fetuses diagnosed by genetic variants were distributed most in increased NT with 10.00% (Additional file 2: Figure S2). The most significant percentage of fetuses diagnosed as single gene disorder was found in skeletal (23.40%), while only 5.32% of fetuses with skeletal defect were diagnosed as a syndrome (Additional file 2: Figure S2).

### Frequency of diagnosis

**Fig. 2:**
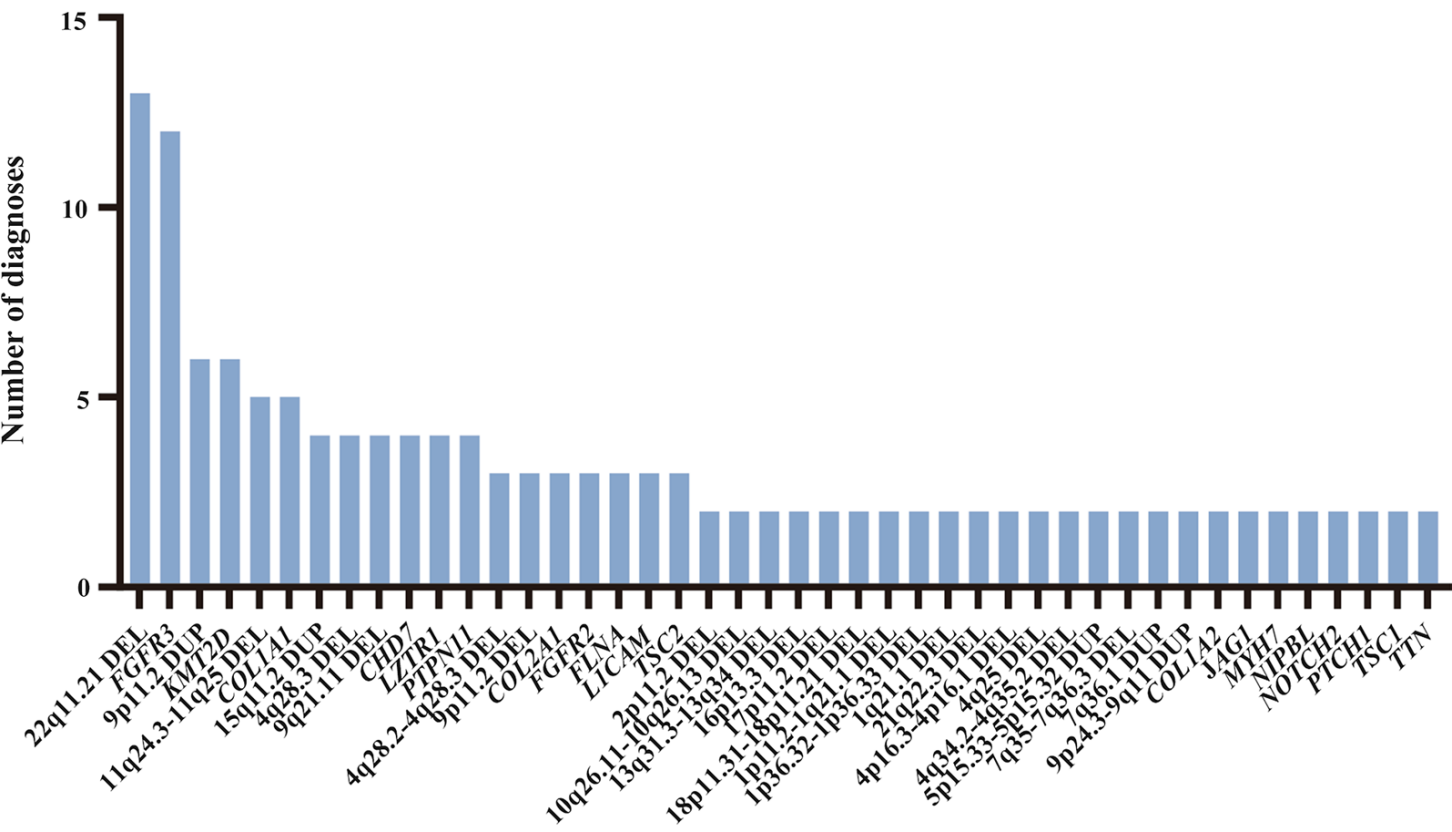
The number of diagnoses of CNVs and genes which emerged variants in fetuses of a total present cohort. Fetuses with double diagnosis of CNVs and variants were counted more than once. *DEL* deletion; *DUP* duplication

Forty-four types of CNVs and variants were diagnosed in the present cohort more than once involving 143 fetuses (14.91%). The most common diagnosis was 22q11.21 deletion in 13 fetuses (1.36%; Fig. 2). 22q11.21 contained a cluster of low copy repeats, and 22q11.21 deletion was reported as the most common recurrent microdeletion in humans, especially reported as DiGeorge syndrome [32]. The second frequent type was *FGFR3* variants, which could cause a skeletal anomaly in 12 fetuses (1.25%; Fig. 2). Subsequently, 9p11.2 duplication and *KMT2D* variants were detected in 6 fetuses (0.62%; Fig. 2). Especially, trisomy 9p was regarded as one of the most common partial trisomies in newborns [33]. 11q24.3–11q25 deletion, most reported as Jacobsen syndrome, and *COL1A1* variants were diagnostic in 5 fetuses (0.52%; Fig. 2). Seven alterations were 3 times diagnosed (0.31%; Fig. 2), and other 25 were diagnosed twice (0.21%; Fig. 2).

### Additional information of double diagnosed fetal

Notably, among all 10 fetuses with double diagnosed, 3 were congenital heart disease, 3 were skeletal anomalies, 2 were fetal hydrops, 1 was facial anomaly and 1 was abnormal of gastrointestinal tract and abdominal wall (Table 3). Fetal C0290 showed generalized skin edema companied with ascites and pleural effusion after ultrasonic scan. Pathogenic CNV in 16p13.3 (33.98 kb deletion) altered *TSC2* and *PKD1* genes that were postulated to contribute to driving ascites and pleural effusion. The pathogenic SNV on *NIPBL* gene was reported to be associated with general fetal hydrops, which was an additional information for prognosis. The double diagnosis would thoroughly demonstrate the genetic pathogenicity of fetuses, which was robustly crucial in the fetus with general symptoms in different anatomical systems.

**Table 3:** Double diagnosis identified in a cohort of fetuses with structural anomalies (increased NT included)

	Prenatal imaging findings	Gene	Location	Consequence	Inheritance
FetalC0247	Facial	*OR4M2*, *OR4N4*, *POTEB3*, *OR4N4C*, *POTEB*	Chr15, 21422120–22429653	1.01 mb duplication	De novo
		*STAG2*	ChrX, 124062902	Missense variant	Hemizygous male fetus maternal inherited
FetalC0290	Hydrops	*TSC2*, *PKD1*	Chr16, 2084905–2118880	33.98 kb deletion	De novo
		*NIPBL*	Chr5, 37007445	Missense variant	De novo
FetalC0309	Skeletal	*FOXD4L6*, *SPATA31A6*, *CBWD6*, *CBWD6*, *CNTNAP3B*	Chr9, 40992379–42569325	1.58 mb duplication	De novo
		*KIF22*	Chr16, 29802813	Missense variant	De novo
FetalC0450	Cardiac	*GGTLC3*, *RIMBP3*, *TSSK2*, *GSC2*, *SLC25A1*, *MRPL40*, *C22orf39*, *CLDN5*, *SEPTIN5*, *GP1BB*, *RTL10*, *TRMT2A*, *CCDC188*, *THAP7*, *SLC7A4*, *TUBA8*, *USP18*, *TMEM191B*, *DGCR6*, *PRODH*, *DGCR2*, *UFD1*, *CDC45*, *TBX1*, *COMT*, *ARVCF*, *DGCR8*, *RANBP1*, *ZDHHC8*, *RTN4R*, *USP41*, *ZNF74*, *SCARF2*, *SERPIND1*, *SNAP29*, *CRKL*, *LZTR1*, *P2RX6*, *LRRC74B*, *ESS2*, *CLTCL1*, *HIRA*, *GNB1L*, *TANGO2*, *DGCR6L*, *KLHL22*, *MED15*, *PI4KA*, *TXNRD2*, *AIFM3*	Chr22, 18108288–21085716	2.98 mb deletion	De novo
		*CHD4*	Chr12, 6587859	Missense variant	De novo
FetalC0497	Cardiac	*FOXD4L6*, *SPATA31A6*, *CBWD6*, *CBWD6*, *CNTNAP3B*	Chr9, 41034878–42569325	1.53 mb duplication	De novo
		*CHD7*	Chr8, 60828661	Splice acceptor variant	De novo
FetalC0759	Skeletal	*OR4M2*, *OR4N4*, *POTEB3*, *OR4N4C*, *POTEB*	Chr15, 21165579–22279173	1.11 mb duplication	De novo
		*BBS5*	Chr2, 169482248	Splice region variant	Homozygous inherited
FetalC0862	Cardiac	*ADAMTS2*	Chr5, 179343692–179345442	1.75 kb duplication	Compound heterozygous inherited
		*ADAMTS2*	Chr5, 179125137	Missense variant	Compound heterozygous inherited
FetalC1438	Hydrops	*GTF2H2C*, *SERF1B*, *SMN2*	Chr5, 69582366–70785650	1.20 mb deletion	De novo
		*FLNB*	Chr3, 58078804	Missense variant	De novo
FetalC1533	Skeletal	*H3-2*, *PPIAL4E*, *FAM72C*, *NBPF15*	Chr1, 143449487–144450895	1.00 mb deletion	De novo
		*FGFR3*	Chr4, 1804392	Missense variant	De novo
FetalC1595	Gastrointestinal tract and AW	*CYP21A2*	Chr6, 32013119–32044190	31.07 kb duplication	Compound heterozygous inherited
		*CYP21A2*	Chr6, 32038507	Missense variant	Compound heterozygous inherited

Data were listed by identification numbers in the experimental lab of Berry Genomics

*AW* abdominal wall

### Syndromic fetuses

**Fig. 3:**
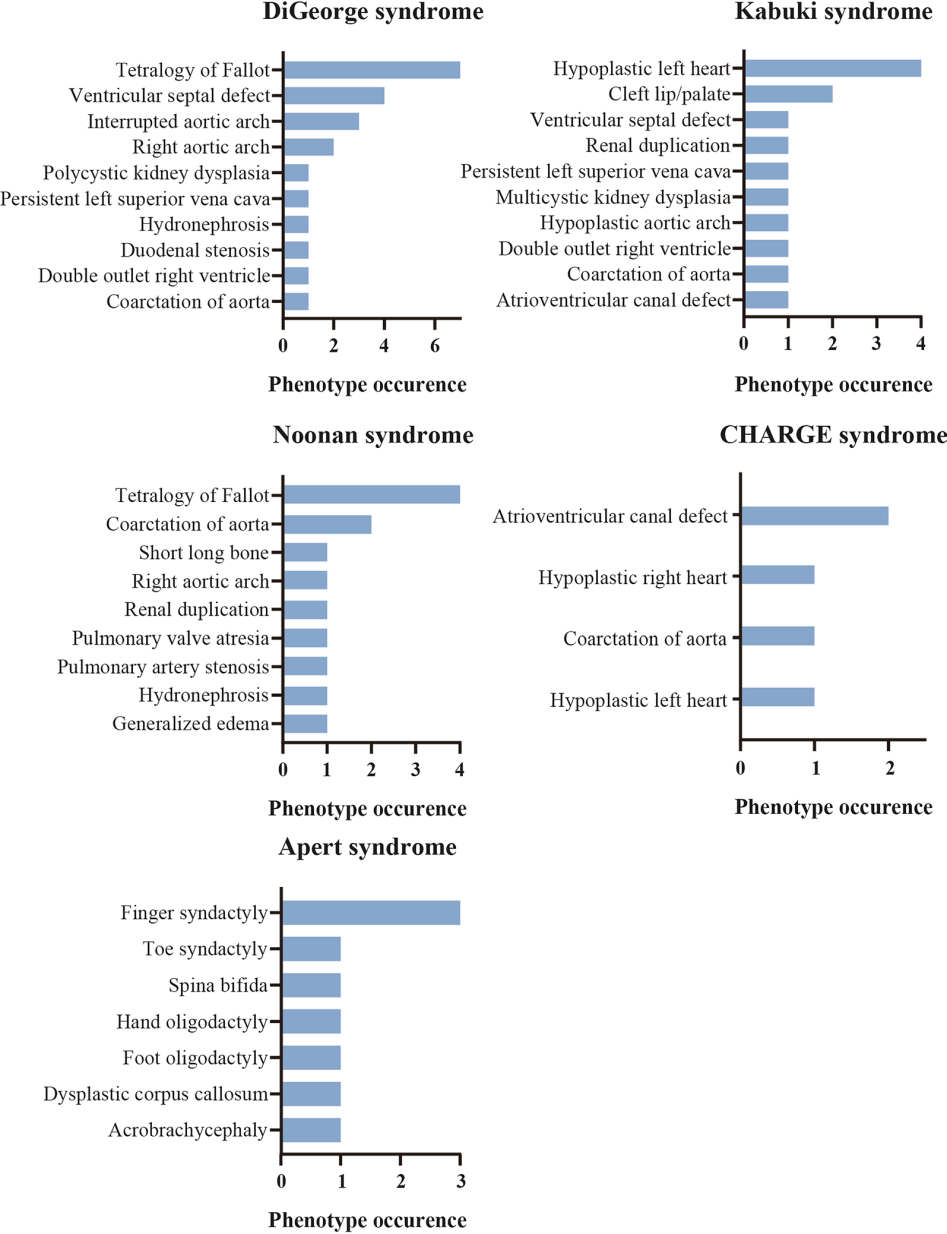
Distribution of phenotype occurrence in the five most diagnosed syndromes in our study cohort. Fetuses with more than one phenotype were counted multiple times

Among all syndromic fetuses, the most 5 frequent syndromes were DiGeorge syndrome (14 fetuses), Noonan syndrome (10 fetuses), Kabuki syndrome (6 fetuses), CHARGE syndrome (4 fetuses), and Apert syndrome (3 fetuses; Fig. 3). Among these 14 diagnosed DiGeorge syndrome fetuses, 7 (50.0%) were screened with tetra of Fallot (TOF) (Fig. 3), which was consistent with previous study [34]. Among 10 fetuses with Noonan syndrome, 4 (40.0%) were scanned with TOF, and 2 (20%) with coarctation of aorta (Fig. 3), both of which were not the most prevalent symptom of Noonan syndrome [35]. In 6 fetuses with Kabuki syndrome, 4 (66.7%) showed hypoplastic left heart (Fig. 3). All 4 fetuses diagnosed with CHARGE syndrome presented various congenital heart defects, including 2 (50.0%) with atrioventricular canal defect, 1 (25.0%) with hypoplastic left heart, and 1 (25.0%) with coarctation of the aorta, as well as 1 (25.0%) with hypoplastic right heart (Fig. 3). All the 3 fetuses with Apert syndrome showed finger syndactyly (3 fetuses, Fig. 3). However, only 1 fetus (33.3%) was observed acrobrachycephaly (Fig. 3), another typical Apert syndrome symptom.

### Fetal outcome

**Fig. 4:**
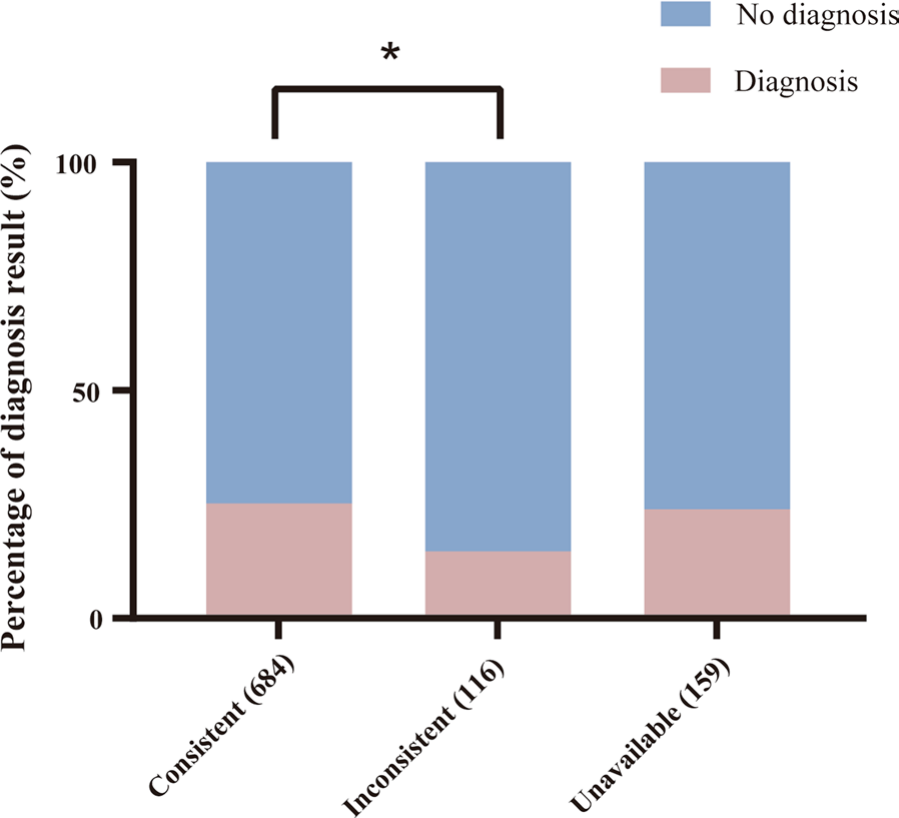
The proportion of diagnosis was associated with the consistency between prenatal ultrasound imaging findings and confirmative phenotype examined by postmortem or postnatal test. A total of 684 fetuses showed consistent intra- and extra-uterine phenotypes, among which 116 fetuses with abnormal prenatal ultrasound findings were found to be normal after birth. Extra-uterine phenotypes were not available in 159 fetuses

Of these 959 fetuses in the present cohort, the fetal outcome was all obtained prior to the test, and a phenotype of postmortem or postnatal examination was also available in 800 fetuses (83.42%) (Fig. 4). The outcome of 719 fetuses (74.97%) was terminations of pregnancy, 7 fetuses (0.73%) experienced neonatal death, and 233 (24.30%) were liveborn. Then we compared the phenotypes of 800 fetuses determined by fetal autopsy or postnatal examination and prenatal ultrasound imaging findings. A total of 684 fetuses showed consistent intra- and extra-uterine phenotypes (Fig. 4). In addition, 116 fetuses with abnormal ultrasound findings showed normal postpartum phenotypes (Fig. 4). Among the 684 fetuses with the consistent phenotype, 172 (25.1%) were diagnosed with genetic alterations. Among the fetuses with inconsistent phenotypes, only 17 were diagnosed with genetic alterations, which was significantly lower than in these with consistent phenotypes (*P*  = 0.0130; Fig. 4).

### Clinical follow-up

All the 959 families were continuously followed up for 6 months after genetic test. Fetal C1768 was screened with skeletal anomaly and the phenotype was confirmed by postmortem. We then detected a maternal inherited 37.16 kb deletion on chromosome 2. However, as the mother showed a normal phenotype, we finally decided not to report this CNV initially. With the progress of follow-up, the same skeletal anomaly was recurrent in the subsequent fetus of C1768 family. Therefore, we re-examined this CNV in fetal C1768 and decided to report as a maternally inherited skeletal anomaly. Furthermore, 12 fetuses were diagnosed with parental inheritance, and another 18 fetuses were diagnosed with paternal or maternal inheritance. A total 43 parents (2.24%) were screened as a carrier of anomalies. In another case, fetal C1101 showed no pathogenic CNVs or variants related to the ultrasound imaging findings, while an *ARID1B* de novo variant was detected. Mental retardation and language delay were observed after birth, which was consistent with the phenotype of Coffin-Siris syndrome mainly caused by mutations of *ARID1B*.

## Discussion

A simultaneous CNV-seq and WES is able to comprehensively detect congenital defects involving CNVs and variants. Double diagnosis would raise the diagnostic yield in the presence of a compound heterozygous state involving CNVs and variants in a trans-phase. In contrast, sequential CMA-ES strategy would partly lead to misdiagnosis in these cases. In our cohort, the proportion of these fetuses was approximately 1.04% of the fetuses, which provided a novel vision into genetic testing of fetal anomalies for those who were not previously diagnosed. Simultaneous CNV-seq and WES could provide additional information for complicated cases, such as fetal C0290 in our study.

Genetic diagnosis at early gestational weeks is crucial for medical control. As an important aspect, attention has been paid to the limitation of samples at early gestational stages. The average input DNA of typical CMA was in a range of 50–100 ng [4]. Additionally, input DNA in a common exome sequencing experimental system was also 50–100 ng [11]. In our study, the sample used for simultaneous CNV-seq and WES analysis was in a range of 60–90 ng with the optimization of our experimental pipeline [26], which yielded a significant decrease in the requirement for initial DNA. Therefore, our genetic testing was much more convenient for fetuses during early gestational weeks or in local hospitals with restricted instruments and equipment.

The TAT of the total pipeline was able to be restricted to at least two weeks after data integration and the parallel analysis. A common strategy for diagnosing fetal congenital anomaly was based on sequential test of karyotype analysis, microarray, and WES in the presence of negative findings in the prior test [11]. The average TAT of each step was 14 days, 14 days, and 14–21 days, respectively. A total of 28–36 days would be consumed on a normal karyotyping analysis. However, it raises a question in those fetuses screened with anomalies in the third trimester, showing a restricted time window for genetic testing. In this study, based on data integration and bioinformatic method, our TAT could compress into 10–14 days, which provides a more applicability strategy in the prenatal phase.

In this study, parental phenotype can merely rely on inquiry or observation. The comprehensive clinical examination of anomalies was hardly available. However, in clinical practice, a lack of parental phenotype, family history, especially the clinical data of siblings may lead to diagnostic ambiguity [36]. A routine of parental clinical examination was also required. On the other side, insufficient phenotype-genotype relationship of prenatal diseases was also a main reason for misdiagnosis. In our cohort, non-benign CNVs and SNVs of *TEKT4* and *CDH18* were repetitively detected. To our best knowledge, few studies have been conducted to reveal the absolute genotype–phenotype relationship of these two genes. Some studies reported the association between *CDH18* gene and congenital heart diseases [37], diabetes mellitus [38], and glioma [39]. Moreover, in randomized phase II clinical trial, Jiang et al. [40] reported that *TEKT4* germline variations in breast cancer were associated with paclitaxel resistance and increased vinorelbine sensitivity. However, their roles in the pathogenesis of certain diseases are not known. In future, more studies are required to further illustrate the potential relationship between genes alternated by CNVs or SNVs and the pathogenesis of certain diseases.

Previous studies indicated that definitive CNVs or genetic variants are associated with certain congenital diseases such as Pelizaeus-Merzbacher disease [41] and congenital heart diseases [42]. Our results elucidated that the etiology of congenital structural defects was extraordinarily complex and heterogeneous consisted of various CNVs and genetic variants locating on a wide-range area of the human genome. In future, more advanced and comprehensive prenatal tests like whole-genome sequencing and long-read sequencing on single molecule real-time sequencing platforms are urgently required to evaluate congenital fetal defects. In addition, the intrauterine stage was a unique growth period of humans featured by rapid development, complex influence factors, and indirectness of symptom detecting [43]. The syndromic congenital defects would be indistinguishable merely depending on prenatal ultrasound screening [43]. The multi-dimension examination can provide guidance on the prognosis and future health care. Simultaneously, the most prevalent prenatal ultrasound findings of syndromes in our cohort were not totally consistent with the reported postnatal phenotypes. On this basis, further prenatal syndrome research was required.

Based on the final diagnosis, 3 inherited CNVs and 30 inherited variants were founded in 31 fetuses, involving 43 parents (12 parental inherited, 19 maternal or paternal inherited, 2.24%) who were confirmed as carriers. Additionally, recurrence was observed in 4 trios involving the second fetus after termination of the first pregnancy in our cohort. Among the 4 fetuses, one showed a maternal inherited mode, while the other 3 showed normal results after combined analysis, which were inferred to be caused by germline mosaicism. These 49 parents, including 43 confirmed carriers and 6 suspected carriers, yielded a proportion of 2.6% in the study cohort, suggesting that a genetic carrier screening of congenital defects was unneglectable.

## Conclusions

With the progress of our experimental and bioinformatic procedure, the novel congenital anomaly testing strategy that simultaneously perform CNV-seq and WES was able to compress the TAT into 10–14 days, and the initial DNA would decrease to 60–90 ng totally. Among the total 959 trios enrolled, median of gestational stage when fetuses were ultrasonic screened with anomaly was 23.6 week. There is indeed a demand for a quicker TAT. Fetuses with cardiac, CNS, facial and multisystem anomaly were the most frequent, which indicating the prevalence of fetal anomaly in Hubei province, China. The diagnostic rate of simultaneously testing was 23.67%, ranging from 9.3 to 41.5% in different phenotypic categories. 10 fetuses were double diagnosis who would be misdiagnosed or losing genetical information in case performing sequential karyotype-CMA-ES strategy. 191 of 227 diagnosed fetuses were de novo. 22q11.21 deletion, *FGFR3* variants and 9p11.2 duplication were the most frequently genetic etiology in our cohort. With a continuously clinical followed up, some cases were detected to be normal by performing newborn examining or autopsy of abortion who were classified as inconsistent group. The diagnostic rate of consistent group was significantly higher than which in the inconsistent group.

## Supplementary Information

**Additional file 1: Figure S1.** Sequencing and bioinformatics analysis pipeline of CNV-seq and WES combined analysis to detect alteration related to congenital structural anomalies. *QC* quality control; *WES* whole-exome sequencing; *CNV* copy number variation; *XHMM* eXome Hidden Markov model.

**Additional file 2: Figure S2.** Distribution of diagnostic yield of the different consequences of alterations in each phenotypic class. CNVs were detailed categorized into exonic CNVs (calling by exonic reads) and other CNVs. Variants were classified into variants related to syndrome and single gene disorder (nonsyndromic). *CNV* copy number variation; *CNS* central nervous system; *AW* abdominal wall; *NT*: nuchal translucency.

**Additional file 3: Table S1.** Phenotypic classification. All included fetuses were categorized into ten phenotypic classes. The classification basis was listed depending on the prenatal imaging findings we detected.

**Additional file 4: Table S2.** The multidisciplinary conference reviewed diagnostic CNVs and genetic variants.

## Data Availability

The data presented in this study are available on request from the corresponding author.

## Abbreviations

CMA: Chromosomal microarray analysis
NGS: Next-generation sequencing
WES: Whole-exome sequencing
SNV: Single-nucleotide variant
InDel: Insertion/deletions
TAT: Turnaround time
MCHHHP: Maternal and Child Health Hospital of Hubei Province
NT: Nuchal translucency
POC: Products of conception
HPO: Human phenotype ontology
ACMG: American College of Medical Genetics and Genomics
CNS: Central nervous system
TOF: Tetra of Fallot
