# Supplementary material for: Clinical efficiency of simultaneous CNV-seq and whole-exome sequencing for testing fetal structural anomalies
Source: J Transl Med. 2022 Jan 3;20:10. doi: 10.1186/s12967-021-03202-9 (PMC8722033; doi:10.1186/s12967-021-03202-9)
Supplement: Supplementary file 1 — Additional file 1: Figure S1. Sequencing and bioinformatics analysis pipeline of CNV-seq and WES combined analysis to detect alteration related to congenital structural anomalies. QC quality control; WES whole-exome sequencing; CNV copy number variation; XHMM eXome Hidden Markov model. [file 12967_2021_3202_MOESM1_ESM.docx]

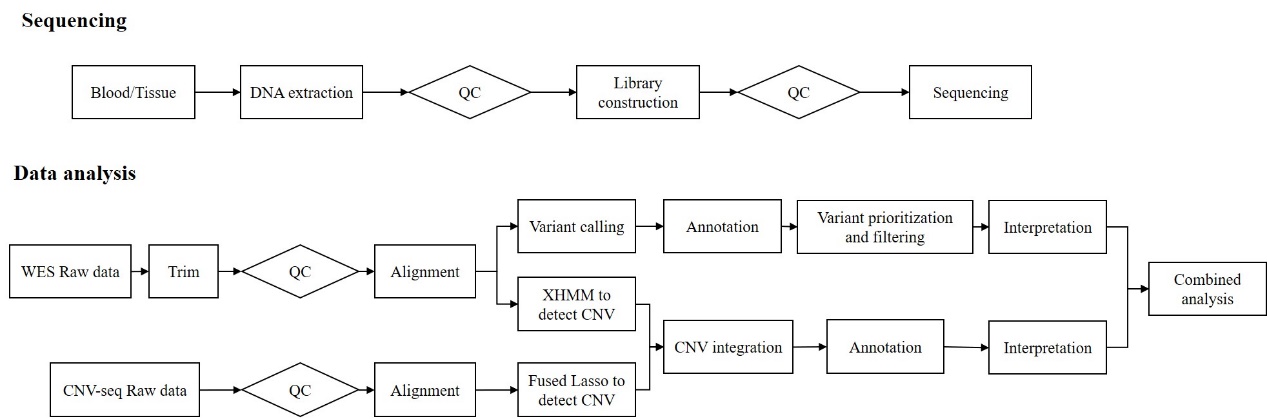


Supplementary Figure 1. Sequencing and bioinformatics analysis pipeline of CNV-seq and WES combined analysis to detect alteration related to congenital structural anomalies. QC: quality control, WES: whole-exome sequencing, CNV: copy number variation, XHMM: eXome Hidden Markov model.
