# Supplementary material for: Clinical efficiency of simultaneous CNV-seq and whole-exome sequencing for testing fetal structural anomalies
Source: J Transl Med. 2022 Jan 3;20:10. doi: 10.1186/s12967-021-03202-9 (PMC8722033; doi:10.1186/s12967-021-03202-9)
Supplement: Supplementary file 2 — Additional file 2: Figure S2. Distribution of diagnostic yield of the different consequences of alterations in each phenotypic class. CNVs were detailed categorized into exonic CNVs (calling by exonic reads) and other CNVs. Variants were classified into variants related to syndrome and single gene disorder (nonsyndromic). CNV copy number variation; CNS central nervous system; AW abdominal wall; NT: nuchal translucency. [file 12967_2021_3202_MOESM2_ESM.docx]

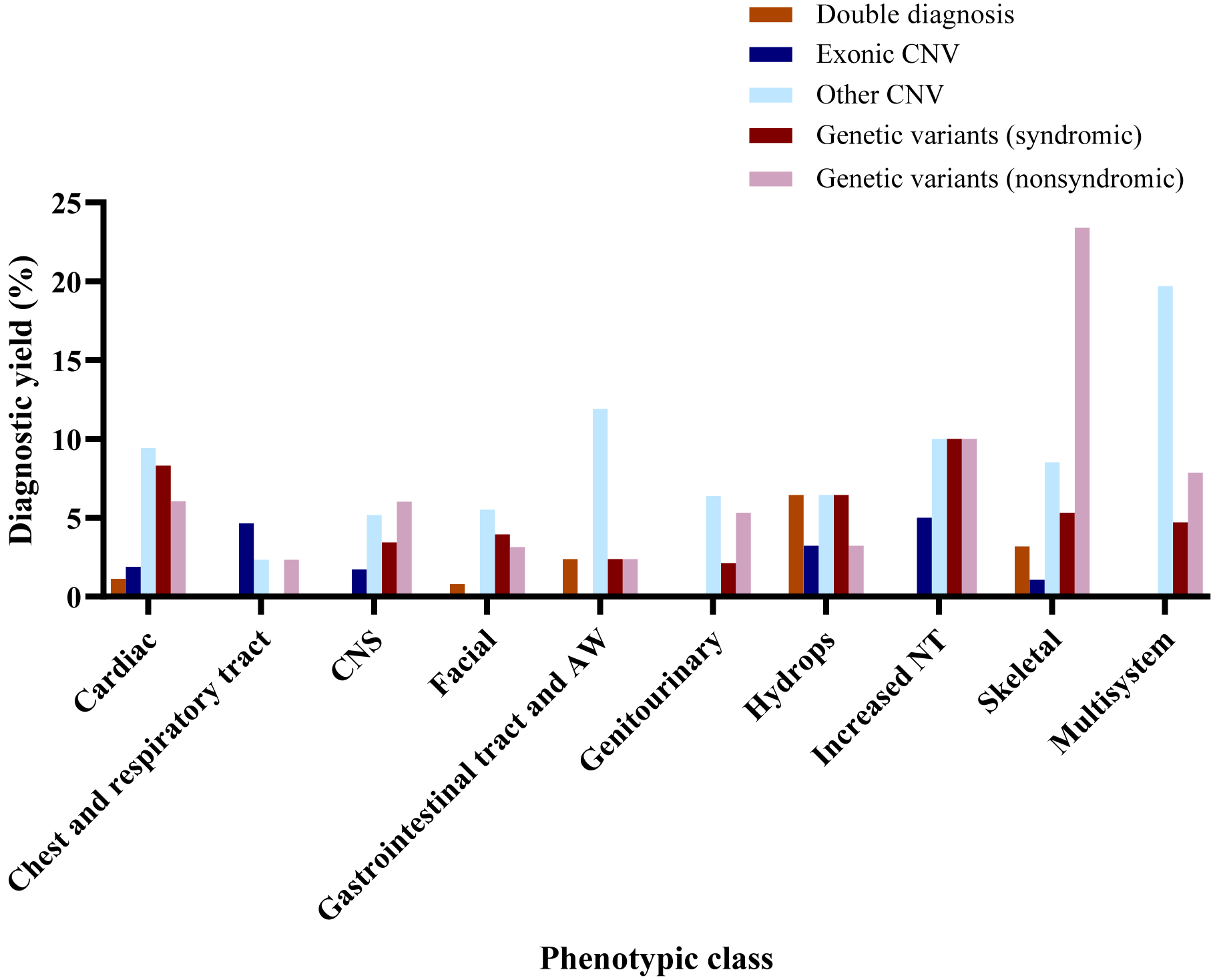


Supplementary Figure 2. Distribution of diagnostic yield of the different consequences of alterations in each phenotypic class. CNVs were detailed categorized into exonic CNVs (calling by exonic reads) and other CNVs. Variants were classified into variants related to syndrome and single gene disorder (nonsyndromic). CNV: copy number variation, CNS: central nervous system, AW: abdominal wall, NT: nuchal translucency.
